# Supplementary material for: MEME-LaB: motif analysis in clusters
Source: Bioinformatics. 2013 May 14;29(13):1696–7. doi: 10.1093/bioinformatics/btt248 (PMC3694638; doi:10.1093/bioinformatics/btt248)
Supplement: Supplementary Data [file supp_btt248_Supplementary_Data.zip › Supplementary_Data/Results_Files/which_gene_ids/cluster9_motif5.html]

Cluster 9 Motif 5


Cluster 9 Motif 5 is found in the following promoters

| Gene ID | Strand | Position\* |
| --- | --- | --- |

|  |  |  |
| --- | --- | --- |
| AT1G01970 | + | 63/500 |
| AT1G04680 | - | 372/500 |
| AT1G09340 | - | 449/500 |
| AT1G12250 | + | 465/500 |
| AT1G20650 | + | 458/500 |
| AT1G21600 | + | 100/500 |
| AT1G29070 | + | 121/500 |
| AT1G32550 | + | 104/500 |
| AT1G36160 | + | 423/500 |
| AT1G49480 | - | 398/500 |
| AT1G60950 | + | 111/500 |
| AT1G67840 | + | 489/500 |
| AT1G71260 | + | 289/500 |
| AT1G72030 | - | 36/500 |
| AT1G74920 | - | 33/500 |
| AT2G17440 | - | 30/500 |
| AT2G30390 | - | 18/500 |
| AT2G33990 | + | 480/500 |
| AT2G34310 | + | 199/500 |
| AT2G38120 | - | 278/500 |
| AT2G42730 | + | 194/500 |
| AT2G47940 | - | 451/500 |
| AT3G01480 | + | 487/500 |
| AT3G06510 | - | 83/500 |
| AT3G07310 | + | 490/500 |
| AT3G09650 | + | 1/500 |
| AT3G10270 | - | 77/500 |
| AT3G17040 | + | 487/500 |
| AT3G23760 | + | 259/500 |
| AT3G24150 | - | 245/500 |
| AT3G25690 | + | 125/500 |
| AT3G26300 | - | 198/500 |
| AT3G28460 | + | 13/500 |
| AT3G29290 | + | 197/500 |
| AT3G47450 | + | 93/500 |
| AT3G54090 | - | 473/500 |
| AT3G63490 | - | 340/500 |
| AT4G09650 | + | 488/500 |
| AT4G30620 | - | 202/500 |
| AT4G32330 | + | 307/500 |
| AT4G33220 | + | 459/500 |
| AT4G35020 | - | 213/500 |
| AT5G09870 | + | 130/500 |
| AT5G11330 | - | 6/500 |
| AT5G12150 | + | 468/500 |
| AT5G13510 | + | 482/500 |
| AT5G13650 | - | 246/500 |
| AT5G17170 | - | 399/500 |
| AT5G22800 | + | 483/500 |
| AT5G23280 | + | 277/500 |
| AT5G27290 | - | 271/500 |
| AT5G51110 | + | 142/500 |
| AT5G54600 | + | 93/500 |
| AT5G55220 | + | 158/500 |
| AT5G62470 | - | 404/500 |

**\*** Overlapping sequences are merged to avoid redundancy. As a result some sequences in the final set may be longer than the specified maximum.

Distribution

+

-

Position values represent number of bases from the 5 prime end of the sequence provided to MEME and the length of that sequence. It is these figures expressed as proportions that are used for statistical analysis.

Probability value of comparison of these values to a linear distribution, p = 0.04

The motif is found 33 times on the + stand and 22 times on the - strand.

Probability value that it is evenly distributed between the two strands, p = 0.089

Move the mouse pointer over the gene ids to highlight the corresponding marker
